# Numerical Study of T-Shaped Micromixers with Vortex-Inducing Obstacles in the Inlet Channels

**DOI:** 10.3390/mi11121122

**Published:** 2020-12-18

**Authors:** Chih-Yang Wu, Bing-Hao Lai

**Affiliations:** Department of Mechanical Engineering, National Cheng Kung University, Tainan 701, Taiwan; bc510608@gmail.com

**Keywords:** microfluidics, T-shaped micromixer, vortex, obstacles, engulfment flow, particle tracking

## Abstract

To enhance fluid mixing, a new approach for inlet flow modification by adding vortex-inducing obstacles (VIOs) in the inlet channels of a T-shaped micromixer is proposed and investigated in this work. We use a commercial computational fluid dynamics code to calculate the pressure and the velocity vectors and, to reduce the numerical diffusion in high-Peclet-number flows, we employ the particle-tracking simulation with an approximation diffusion model to calculate the concentration distribution in the micromixers. The effects of geometric parameters, including the distance between the obstacles and the angle of attack of the obstacles, on the mixing performance of micromixers are studied. From the results, we can observe the following trends: (i) the stretched contact surface between different fluids caused by antisymmetric VIOs happens for the cases with the Reynolds number (*Re*) greater than or equal to 27 and the enhancement of mixing increases with the increase of Reynolds number gradually, and (ii) the onset of the engulfment flow happens at Re≈125 in the T-shaped mixer with symmetric VIOs or at Re≈140 in the standard planar T-shaped mixer and results in a sudden increase of the degree of mixing. The results indicate that the early initiation of transversal convection by either symmetric or antisymmetric VIOs can enhance fluid mixing at a relatively lower *Re*.

## 1. Introduction

Microfluidic mixing has wide applications in biochemical reactions, chemical synthesis and biological analysis [1,2,3]. Mixing in microfluidic devices is challenging since typical flows in microfluidic devices are laminar in nature and mixing in laminar flows relies mainly on molecular diffusion. Therefore, increasing the interface or shortening the diffusion length between different fluids by handling fluid flows within the mixing channel is of great importance for enhancing mixing in microfluidic devices. The reviews of the related literature reveal that various micromixers, broadly categorized as either active or passive types, have been developed and examined. Passive micromixers utilize system geometry to create favorable hydrodynamics for enhancing mixing and so do not require external energy, except that used to drive the flows. Although the mixing efficiency of active micromixers is better than that of passive micromixers, it is simpler to fabricate passive micromixers and easier to integrate them with microfluidic systems. Thus, passive micromixers have been developed widely [1,2,3].

Most passive micromixers include a T or Y junction for the confluence of the fluids to be mixed, and a straight mixing channel with square or rectangular cross-section [4,5,6,7,8,9,10,11,12,13,14] or a mixing channel with modified geometry aiming at enhancing the mixing of fluids [15,16,17,18,19,20,21,22,23,24,25,26,27,28,29,30,31]. The T-, Y- or arrow-shaped mixer with a straight mixing channel has been reported to show the so-called engulfment flow and good mixing at a higher Reynolds number (*Re*) defined as $Re=v_{m}\;d_{h}\rho /\mu$, with $\nu_{m}$, $d_{h}$, ρ and μ denoting the mean flow speed in the mixing channel, the hydraulic diameter of the mixing channel, the density and the dynamic viscosity of the fluid, respectively. Engler et al. [5] have found that the resulting flow in the mixing channel of a planar T-shaped mixer can be characterized by three flow regimes during steady flow: so-called “stratified” flow, “vortex” flow and “engulfment” flow. They showed that the breakup of symmetry in the flow field at a higher Reynolds number occurred, resulting in the so-called engulfment flow, which was characterized by some fluid from one side reaching beyond the centerline of the micro-T-shaped mixer to engulf the fluid from the other side. It has been found that, in a standard planar T-shaped mixer with two inlets with square cross-section, and a mixing channel of equal combined area where two-opposing planar channel streams join and turn through 90 degrees into the mixing channel, the symmetry breaking of the flow field configuration in the mixing channel starts at a Reynolds number between 138.6 and 140 [7]. The influence of the volume flow rates has been well investigated. Schikarski et al. reported an experimental–computational study of a T-shaped mixer for Reynolds numbers up to 4000 [12]. Besides the Reynolds number, the flow patterns and mixing performance may vary with other flow or geometric parameters. Soleymani et al. proposed a dimensionless number to describe the dependence of the flow regime inside the T-micromixer on the flow rate, aspect ratio and hydrodynamic diameter ratio [8]. Galletti et al. investigated the effect of inlet velocity distributions on the mixing performance [11]. Karvelas et al. investigated the effect of the angle of the Y-shaped micromixer and the effect of different inlet velocity ratios [13]. Recently, Camarri et al. presented an overview study on the effects of the Reynolds number, aspect ratios and mixing angle on the mixing performance of a T-shaped micromixer [14].

Since mixing in a T-, Y- or arrow-shaped mixer with a straight mixing channel is ineffective at lower values of *Re*, various modifications in the geometry of mixing channel have been developed and investigated. These modified mixing channels include introduction of obstacles [15,16] at high Reynolds numbers, serpentine and/or converging–diverging channels [17,18,19,20] for *Re* in the range 10–100, and patterned groove microchannels at low Reynolds numbers [21,22]. The other types of micromixers adopted serial lamination [23], split-and-recombine (SAR) channels [24,25], two-layer crossing channels [26], channels with cross-sections other than square or rectangular [30,31] and channel modifications based on combined different mixing mechanisms [27,28,29,31]. Most existing research has investigated the geometric modification of the mixing channel from the junction to the outlet for enhancing fluid mixing in micromixers, while only a few works take the effects of inlet channel modification into account. Gigras and Pushpavanam have proposed and analyzed the fluid mixing in a micromixer with a curved inlet channel which exploits the early induction of transversal vortices in the inlet channel to enhance mixing [19]. Sultan et al. studied flow behavior and mixing in mixers with T-shaped jet inlets for different geometrical parameters [32,33]. Three-dimensional (3D) T-type micromixers with non-aligned inlets were proposed for convective mixing enhancement [34,35,36]. Other than the geometric modification of the mixing channel, droplet micromixers [37], micromixers using ultra-hydrophobic surfaces [38] and more designs have been proposed by many researchers (see References [1,2,3] for reviews of these) to improve the mixing quality in passive micromixers.

**Figure 1:**
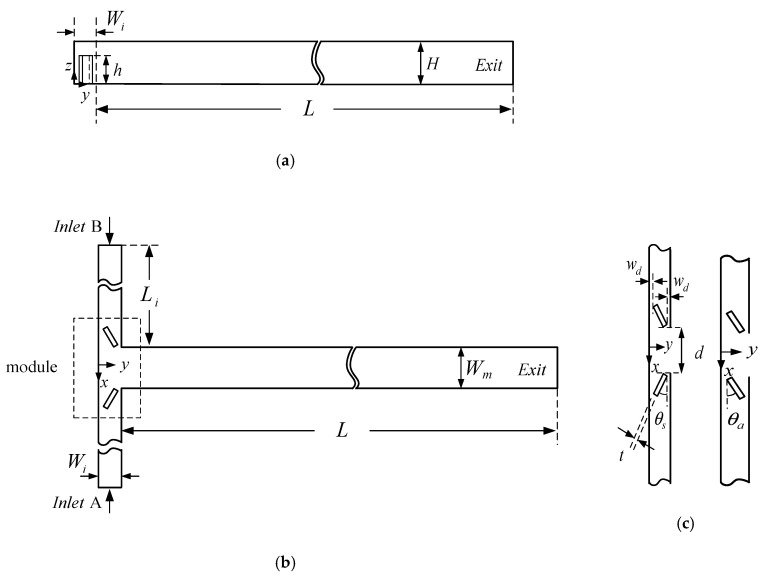
Schematic diagram and geometric parameters of T-shaped micromixers with vortex-inducing obstacles (VIOs) in the inlet channels: (**a**) side view, (**b**) top view, (**c**) inlet channel with symmetric VIOs (**Left**) and that with as antisymmetric VIOs (**Right**).

A new approach for inlet flow modification by adding obstacles protruding into the flow at an angle of attack in the inlet channels of a T-shaped micromixer is proposed and investigated in this work. The obstacles with a height less than the channel height may induce vortices at the entrance of the mixing channel at a relatively lower Reynolds number and are called the vortex-inducing obstacles (VIOs) in this work. In light of the above-mentioned considerations, it is desirable to explore if the early introduction of vortices before the entrance of the mixing channel can lead to a fundamental improvement in fluid mixing of a T-shaped micromixer. The present work focuses on the effects of geometric modifications of the inlet channel on mixing and does not consider the geometric modification of the mixing channel, such as those proposed in the literature [15,16,17,18,19,20,21,22,23,24,25,26,27,28,29,30,31], or active strategy of mixing, such as the application of magnetic field to the mixing of contaminated water in one stream and water with the magnetic particles in the other stream [39]. The VIOs may be symmetric or antisymmetric, as shown in Figure 1. Numerical simulation has been performed to investigate the mixing behavior and flow characteristics for selected values of geometric parameters of the added VIOs in the inlet channels at different flow rates. To study the effects of the early induction of transversal convection by the VIOs, comparisons of the mixing performance of the proposed mixer with either type of the VIOs and that of a standard T-shaped mixer without VIOs are made. The results indicate that the early initiation of transversal convection by either symmetric or antisymmetric VIOs can enhance fluid mixing at a relatively lower *Re*.

## 2. Mixer Geometry, Governing Equations and Computational Procedure

The present micromixers consist of two inlets and a mixing channel, where two opposing streams of different fluids join and turn through 90 degrees into the mixing channel leading towards the outlet. A pair of oblique VIOs are mounted on the bottom of the inlet channels. The pair of VIOs protrudes into the flow at an angle of attack ($\theta_{s}$ or $\theta_{a}$) symmetrically or anti-symmetrically and has the height of h<H, as shown in Figure 1. Here, *H* is the channel height. The micromixers can be fabricated by using an easy two-step lithography process. There are a very large number of geometrical parameters influencing the performance of the micromixers. The effects of the widths and height of the inlet and mixing channels on fluid mixing have been investigated systematically [8]. The present study focuses on examining the influence of the arrangement of the VIOs, including the distance between the VIOs (*d*) and the angle of attack ($\theta_{s}$ or $\theta_{a}$), on fluid mixing; in the meantime, we set the value of H at 120 μm, the width of the mixing channel $W_{m}=2H$, the width of the inlet channels $W_{i}=H$, the thickness of the VIOs t=0.25H, the height of the VIOs h=0.75H, the length of the inlet channel $L_{i}=8H$, the length of the mixing channel L=15H, the distance between each obstacle and its neighboring sidewall $w_{d}=0.25H$.

The fluid was assumed to be Newtonian, isothermal and incompressible, and the corresponding governing equations of the flow field are the continuity and the Navier–Stokes equations, as follows:

(1) $$\nabla \cdot {\vec{v}}^{*}=0,$$

(2) $$\left({\vec{v}}^{*}\cdot \nabla \right){\vec{v}}^{*}=-\nabla p^{*}+\frac{1}{Re}\nabla^{2}{\vec{v}}^{*},$$

where, ${\vec{v}}^{*}=\frac{\vec{v}}{v_{m}}$ and $p^{*}=\frac{p-p_{0}}{\rho v_{m}^{2}}$, with $\vec{v}$, p and $p_{0}$ denoting the velocity vector, the pressure and the atmosphere pressure, respectively. In this work, we consider equal flow rate at both inlets, and so the mixing ratio is 1:1. The inlet velocity is set to be a fully developed laminar velocity profile [40]. The no-slip condition is imposed on all solid walls and the pressure at the exit is set to be 1 atm. The concentration field is described by the convective–diffusive equation:

(3) $$\left({\vec{v}}^{*}\cdot \nabla \right)c^{*}=\frac{1}{ReSc}\nabla^{2}c^{*}$$

where $c^{*}=\frac{c-c_{B}}{c_{A}-c_{B}}$, with c denoting the molar concentration per unit volume and the subscripts *A* and *B* denoting the inlet A and the inlet B, respectively. Sc=μ/ρD is the Schmidt number, with *D* denoting the diffusion coefficient. The nondimensional molar concentration $c^{*}$ takes a quantity from 0 to 1. The solid walls are set to be impermeable. The fluids entering the two inlets are a low-concentration solution of Rhodamine B in deionized (DI) water with a diffusion coefficient $3.6\times 10^{-10}$ $m^{2}{\;s}^{-1}$ [41] and pure DI water. Because of the low concentration of the solution, the influence of fluorescent on the fluid behavior can be neglected [5], and so the fluid properties considered are set to be those of the DI water with density, ρ=997 ${\mathrm{kg}\;m}^{-3}$, and viscosity, μ= 0.00097 ${\mathrm{kg}\;s}^{-1}m^{-1}$. Therefore, the Schmidt number equals 2700.

**Figure 2:**
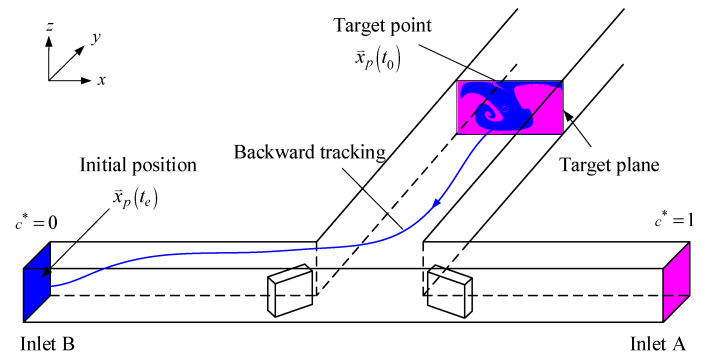
Three-dimensional (3D) micromixer geometry and the sketch of backward fluid particle tracking.

The pressure and the velocity vector are solved by a grid-based scheme, using the software CFD-ACE+ (CFD Research Corporation, Huntsville, AL, USA). The SIMPLEC (Semi-Implicit Method for Pressure Linked Equations-Consistent) algorithm is used for pressure-velocity coupling and the spatial difference is carried out with the second-order upwind scheme with limiter. When the relative residual of each variable is low to $10^{-5}$, the solution is regarded as converged. In this work, we are mainly interested in the effect of transverse convection on fluid mixing, which typically dominates at a high Reynolds number. For such cases with Sc=2700, the value of the Peclet number (Pe=ReSc) of the flow in the mixer may be high. The grid-based solutions of the convective–diffusive equation suffer from the false numerical diffusion at a high Peclet number [42]. Therefore, to simulate fluid mixing in the proposed micromixers under various conditions, we adopt the particle tracking method with an approximation diffusion model (ADM), which is virtually free of numerical diffusion in high-Peclet-number flows and takes less computation time than the random-walk particle-tracking method [43,44]. To determine the non-diffused concentration field, we undertake the particle-tracking simulation, which is illustrated by an example shown in Figure 2. The target planes are evenly divided into $\left(N_{xt}-1)\times (N_{zt}-1\right)$ grid cells and a massless particle is assigned to each grid cell. Then, these particles are backward tracked from the target plane to the two inlets. The advection displacement of a particle can be calculated by using a selected small advection time step and interpolating the velocity data at grid points, which are available from the solution by the software CFD-ACE+. The boundary condition of the concentration field can be accommodated by suitable particle reflections from the boundary [45]. The displacements are repeated until the particle crosses either of the two inlets. The details of the backward particle-tracking simulation were reported by Kuo et al. [44]. Next, the diffused concentration field is obtained by solving the approximation diffusion equation with the non-diffused concentration field as the initial condition. This is a posterior treatment of molecular diffusion proposed by Matsugana et al. [43]. A discretization scheme based on a five-point formula is employed to solve the diffusion equation.

To quantitatively analyze the mixing performance, the degree of mixing at each cross-section of the micromixer is evaluated by:

(4) $$M=1-\frac{\sigma }{\sigma_{0}},$$

where σ denotes the variance of the concentration at a transverse cross-section, defined as:

(5) $$\sigma^{2}=\frac{1}{N_{s}}\sum_{n=1}^{N_{s}}{\left(c_{n}-\bar{c}\right)}^{2}$$

with $N_{s}$ denoting the total number of sampling, $c_{n}$ the concentration at a position on the cross-section considered, $\bar{c}$ the average of $c_{n}$ and $\sigma_{0}^{}=\sqrt{\bar{c}\left(1-\bar{c}\right)}$ the variance of the concentration in the completely unmixed state [46]. In this work, we estimate the mixing efficiency of a micromixer by calculating the degree of mixing at the exit cross-section. The total number of sampling is the number of grid cells on the target plane, $\left(N_{yt}-1)\times (N_{zt}-1\right)$, for the particle-tracking simulation with an ADM.

## 3. Results and Discussion

**Figure 3:**
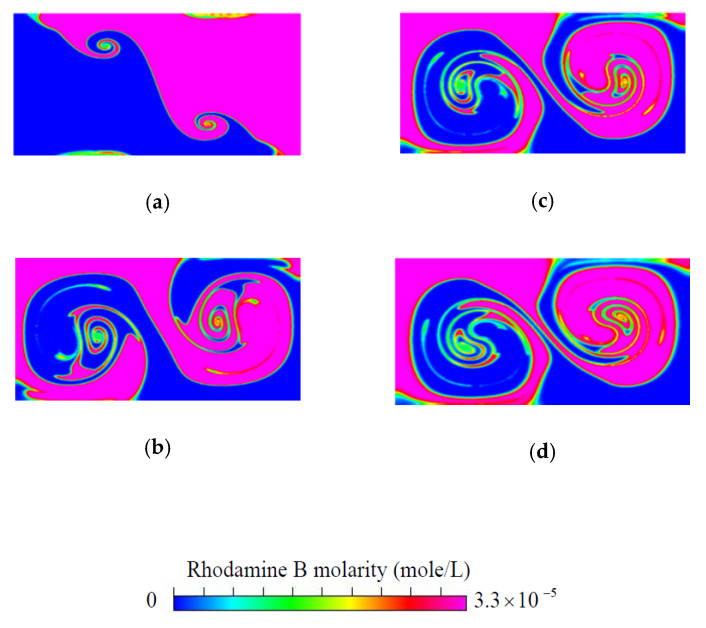
Concentration distributions on the cross-section at (**a**) y=300 μm, (**b**) y=1500 μm, (**c**) y=2700 μm, (**d**) y=3900 μm in a standard planar T-shaped mixer with H=300 μm at $Re^{\prime }=150$ and Sc=3200.

**Figure 4:**
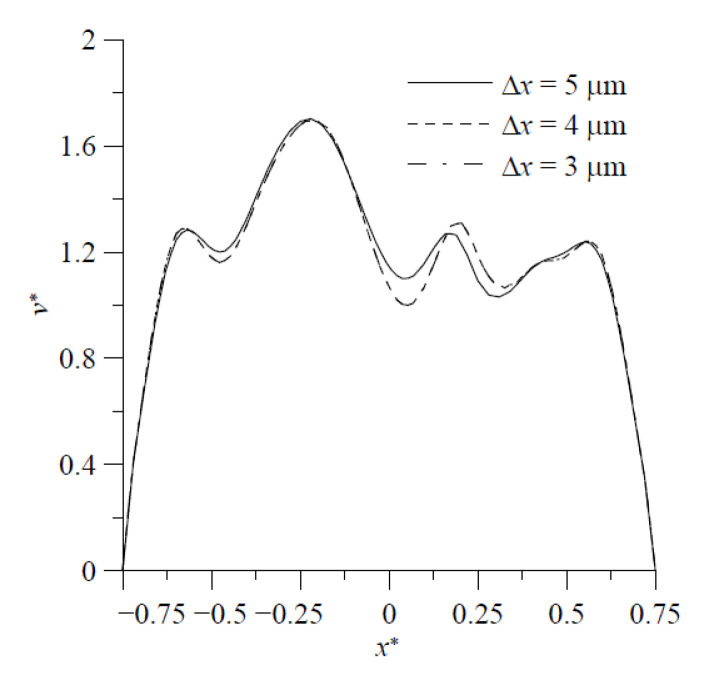
Profiles of velocity component *v* along the x direction on the middle horizontal on the cross-section at y=120 μm obtained by using various grid sizes.

**Figure 5:**
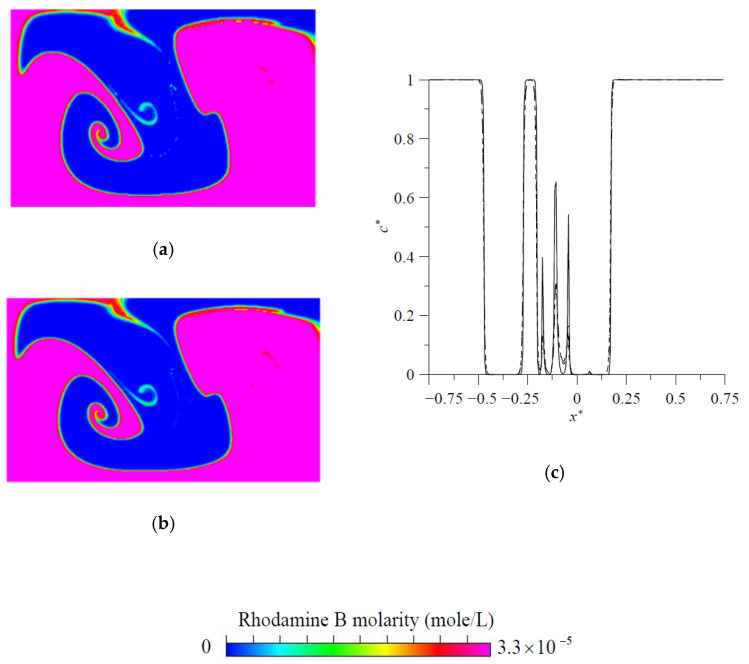
Concentration distributions on the cross-section at y=360 μm obtained by the particle tracking method with an ADM: concentration distributions on the cross-section by using (**a**) *N_xt_* × *N_zt_* = 360 × 180 particles and (**b**) *N_xt_* × *N_zt_* = 480 × 240 particles, (**c**) concentration profiles along the middle horizontal on the cross-section at y=360 μm by using *N_xt_* × *N_zt_* = 240 × 120 particles (──; *N_xt_* × *N_zt_* = 360 × 180 particles (− − − −); *N_xt_* × *N_zt_* = 480 × 240 particles (— - —).

For the purpose of validation, the results of fluid mixing in a standard planar T-shaped mixer with H=300 μm at $Re^{\prime }=Hv_{m}\rho /\mu =150$ and Sc=3200 [43] are considered first. The distributions of concentration on the cross-section at y=1500 μm, obtained by the particle-tracking method with an ADM, are shown in Figure 3. It is easy to see an excellent agreement of present results obtained by using $N_{y}\times N_{z}=80\times 40$ grids in the velocity solution and $(N_{yt}-1)\times (N_{zt}-1)=$600×300 particles in the concentration simulation with those reported by Matsunaga et al. [43]. Then, grid size sensitivity tests were performed for mixing flow at Re= 200 in a proposed mixer with H=120 μm, $\theta_{s}$= 20°, d=3H, h=0.75H, t=0.25H and $w_{d}=0.25H$. The velocity components $v^{*}=v/v_{m}$ along z=150 μm on the cross-section at y=120 μm obtained by using the grid sizes 3 μm, 4 μm and 5 μm are shown in Figure 4. The discrepancy between those obtained by using the grid sizes of 3 μm and 4 μm is quite small. Particle number sensitivity tests were performed for mixing flow at Re= 150 in the same mixer. The distributions of concentration in the present micromixer on the cross-section at y=360 μm, obtained by the particle-tracking method with an ADM using different total numbers of particles, are in excellent agreement, as shown in Figure 5a,b. Figure 5c shows that the distributions of concentration on the cross-section at y=360 μm obtained by using particle numbers 450 × 225 and 600 × 300 almost overlap with each other. Since the geometry of the present mixers are more complicated than a standard planar T-shaped mixer, we use the grid size of 3 μm and 600 × 300 particles launched from a target plane in the following simulation.

**Figure 6:**
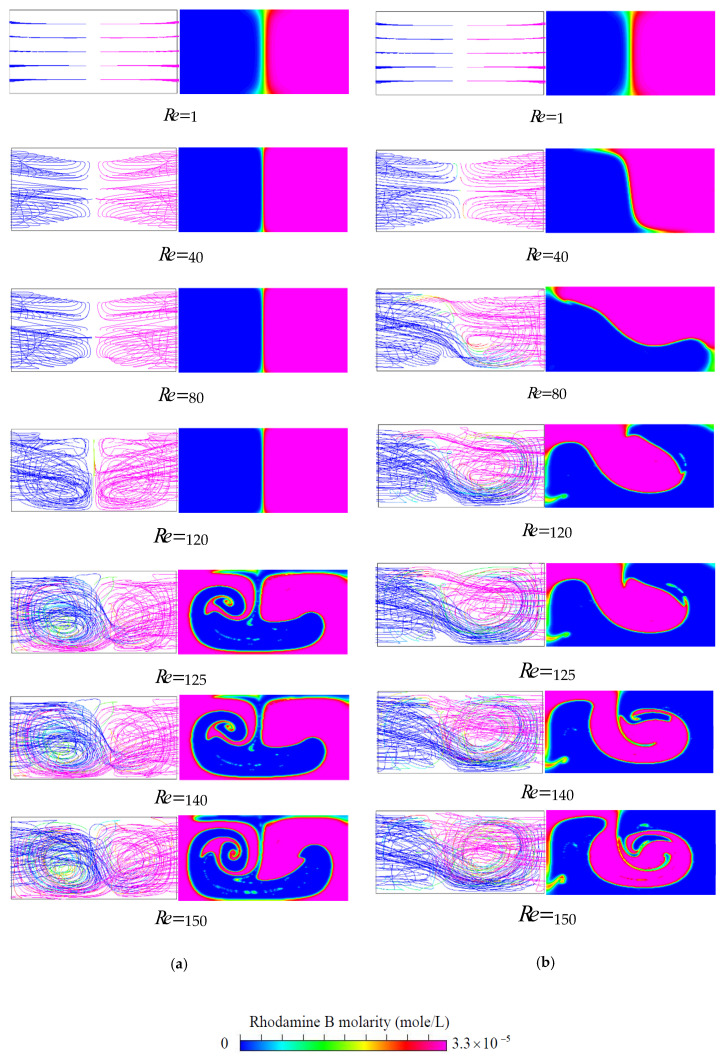
Projected path lines in the T junctions for different mass flows from the left and right inlet channels and the concentration distributions on the cross-section at the exit of (**a**) the mixer with symmetric VIOs and (**b**) the mixer with antisymmetric VIOs. View is into the mixing channel.

The representative path lines and the concentration distributions shown in Figure 6 give an overview of the flow and mixing taking place in the proposed T-shaped micromixers with VIOs in the inlet channels for some selected values of Reynolds numbers between 1 and 150. The selected geometrical parameters associated with the VIOs are h=0.75H,t=0.25H,$w_{d}=0.25H$,$\theta_{s}$= 20° and $\theta_{a}$= 30°. It is easy to see that the flow and mixing in the mixer with symmetric VIOs and those in the mixer with antisymmetric VIOs exhibit different patterns.

**Figure 7:**
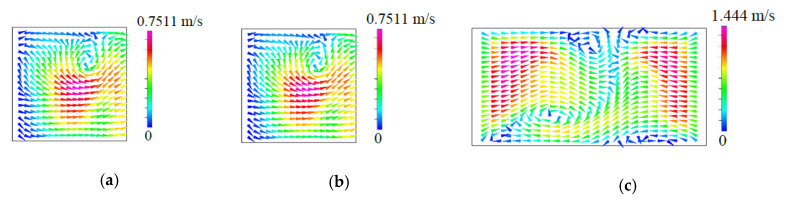
*yz*-projection of the velocity vectors on the cross-sections at (**a**) *x = H*, (**b**) *x = −H*, (**c**) *xz*-projection of the velocity vectors on the cross-sections at *y = H* for the case with *Re = 140*, $\theta_{s}$ = 20°, *h* = 0.75*H*, *t* = 0.25*H* and $w_{d}=0.25H$.

The left and the right columns of Figure 6a show the flow characteristics and the mixing behavior for fluid flow in the T-shaped micromixers with symmetric VIOs, respectively. There is no formation of any vortex flow due to low inertia force in the fluid streams at Re=1, while the path lines of the cases with Re=40~120 show symmetric vortex flows with respect to the plane x=0. Thus, the two portions of fluid entering from the two inlet channels remain segregated and mixing occurs only through diffusion. The flow regime of diffusive mixing is characterized by its flow pattern with single reflection symmetry with respect to the plane x=0, which is different from the double reflection symmetry found in the flow of planar T-shaped micromixers with square or rectangular cross-section [5,7,8,9,10,14]. The double reflection symmetry even appears in the flow regime of diffusive mixing for flow in planar T-shaped micromixers with non-fully developed flow conditions [11]. The diffusion zone around the vertical plane x=0 becomes thinner as the *Re* becomes larger and the mixing states are not desirable yet because of the slow diffusion mechanism. When the *Re* further increases and reaches a critical value, the onset of the so-called engulfment regime takes place. In this flow regime, the flow pattern becomes asymmetric and each inlet fluid stream reaches the opposite sides of the plane x=0, as indicated by the path lines shown in Figure 6a and the velocity vectors shown in Figure 7. Similar to fluid mixing in planar T-shaped micromixers [5,6,7,8,9,10,14,32,33], a sudden increase of the degree of mixing can be observed which originates from the intertwining of the path lines. However, the asymmetric flow pattern shown in Figure 7c is different from the rotational-symmetric flow [7], which appears in the steady engulfment regime of flow in a planar T-shaped micromixer. Although the vortical flow induced by the symmetric VIOs is the same in the two inlet channels, its distribution is not symmetric, as shown in Figure 7a, b. Thus, Figure 7c exhibits the asymmetric flow pattern at a location in the mixing channel for the steady engulfment regime.

**Figure 8:**
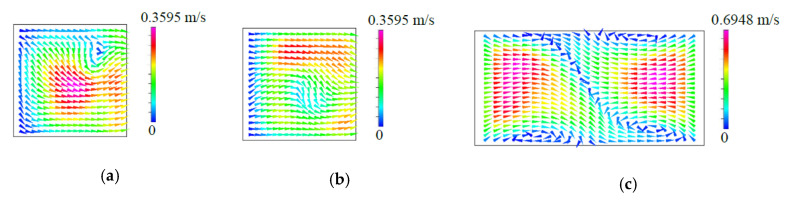
*yz*-projection of the velocity vectors on the cross-sections at (**a**) *x = H*, (**b**) *x = −H*, (**c**) *xz*-projection of the velocity vectors on the cross-sections at *y = H* for the case with *Re* = 80, $\theta_{a}$ = 30°, *h* = 0.75*H*, *t* = 0.25*H* and $w_{d}=0.25H$.

By contrast with fluid mixing in the T-shaped micromixers with symmetric VIOs, from the path lines and the concentration distributions shown in Figure 6b for fluid flow in the T-shaped micromixers with antisymmetric VIOs, we can observe the following trends: the symmetry of the path lines and the concentration distributions holds in the flow at Re=1, the distortion of the interface and the interaction of the two vortices appears at the cases with Re≥40 and the enhancement of fluid mixing by the stretched contact surface of the fluids gradually increases with the increase of the *Re*. This improved mixing is attributed to the asymmetric creation of secondary flow by antisymmetric VIOs in the opposing inlet channels, as shown in Figure 8. The early induced asymmetric lateral convection in the inlet channels breaks up the symmetry of the flow field and augments the contact area between the two streams entering from the two inlet channels, and so enhances fluid mixing at a relatively lower Reynolds number, as shown in Figure 6b. The improved mixing is different from the mixing enhancement caused by the symmetry-breaking bifurcation of fluid flow in the T-shaped micromixer with symmetric VIOs at a higher Reynolds number. A 3D T-mixer with the inlet channels located at different horizontal levels can generate vertical flow into the mixing channel to improve fluid mixing [34,35,36], and so it also runs without the benefit of the symmetry-breaking bifurcation of fluid flow in the planar T-shaped micromixer.

**Figure 9:**
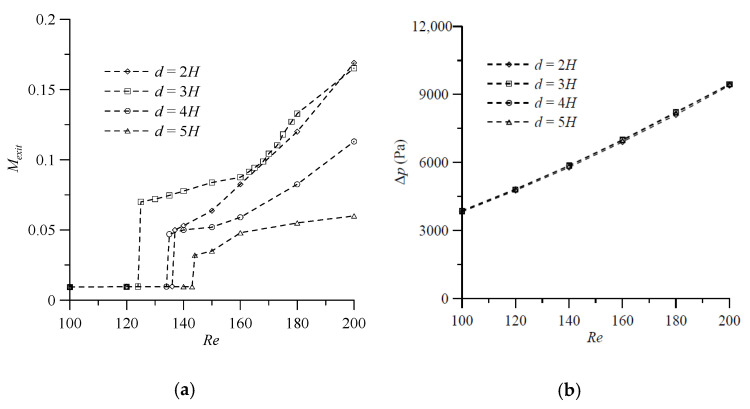
Effect of distance between the symmetric VIOs on (**a**) the degree of mixing and (**b**) the pressure drop in the mixers with $\theta_{s}$ = 20°, *h* = 0.75*H*, *t* = 0.25*H* and $w_{d}=0.25H$ for various Reynolds numbers.

Before quantitatively comparing the mixing performance of the proposed mixer with symmetric VIOs, the proposed mixer with antisymmetric VIOs and a standard T-shaped mixer, we examine the simulation results for cases with various values of the distance between the VIOs (d) and the angle of attack ($\theta_{s}$ or $\theta_{a}$) to investigate the influence of these geometrical parameters and to select the appropriate values of these geometric parameters for efficient mixing. Mixing at a microfluidic junction is strongly dependent on *Re*. Thus, this section reports the results from numerical simulations carried out over a range of *Re* to examine the performance of the proposed mixer with VIOs. First, to make a comparative study of influence of the distance between the symmetric VIOs, we choose four values of the distance, d=2H, 3H, 4H and 5H, and the other geometrical parameters associated with the obstacles are kept same. The corresponding values are $\theta_{s}$ = 20°, h=0.75H, t=0.25H and $w_{d}=0.25H$. The conducted numerical simulation for fluid mixing of a T-shaped mixer with symmetric VIOs in the Reynolds number range from 100 to 200 allows for identifying the value of *Re* corresponding to the transition from the vortex flow regime to the engulfment flow regime. The degree of mixing at the exit ($M_{exit}$) shows a significant increase at such a value of *Re* and increases with the increase of *Re* as the *Re* increases beyond the critical value of *Re* corresponding to the transition, as shown in Figure 9a. It is worth noting that for the cases with d=2H, 3H and 4H, the degree of mixing increases suddenly at a Reynolds number less than the critical value of *Re* reported in the literature for mixing in a standard T-shaped mixer [7]. Moreover, the critical value of *Re* corresponding to the transition for the case with d=3H is the smallest and the degree of mixing of the case with d=3H is greater than or equal to that of the case with d=2H, 4H or 5H, except that the $M_{exit}$ of the case with d=2H is a little bit greater than that of the case with d=3H at *Re* = 200. The pressure drop between the mixer inlet and outlet (Δp) monotonically increases with the increase of the Reynolds number in the cases considered. The influence of the distance between the VIOs on the pressure drop is very small and the transition from the vortex flow regime and to the engulfment flow regime has no effect on the pressure drop, as shown in Figure 9b.

**Figure 10:**
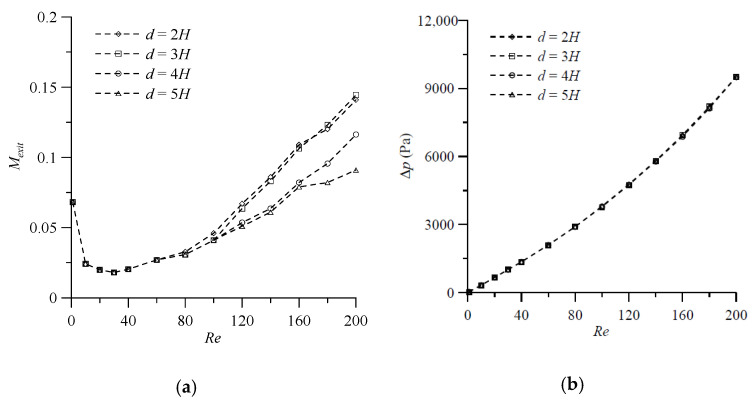
Effect of the distance between the antisymmetric VIOs on (**a**) the degree of mixing, and (**b**) the pressure drop in the mixers with $\theta_{a}$ = 30°, *h* = 0.75*H*, *t* = 0.25*H* and $w_{d}=0.25H$ for various Reynolds numbers.

Similarly, to make a comparative study of influence of the distance between the antisymmetric VIOs, we choose four values of the distance, d=2H, 3H, 4H, and 5H, and the other geometrical parameters, $\theta_{a}$ = 30°, h=0.75H, t=0.25H and $w_{d}=0.25H$, are kept constant. From the preliminary results shown in Figure 6b for fluid flow in the T-shaped micromixers with antisymmetric VIOs, we know that the distortion of the interface and the interaction of the two vortices may appear at a *Re* less than 100. Thus, the simulation results for fluid mixing of a T-shaped mixer with antisymmetric VIOs are examined in a wider Reynolds number range from 1 to 200. Figure 10a reveals that the degrees of mixing of the four cases considered increase gradually as the *Re* increases beyond 30. The enhancement of fluid mixing is caused by the early induction of transversal convection in the inlet channel, which stretches the contact surface of the fluids. The degree of mixing of the case with d=2H and that of the case with d=3H are comparable and they are larger than that of the case with d=4H or 5H. The effect of the early induction of transversal convection by antisymmetric VIOs in the inlet channel on fluid mixing increases with the increase of *Re*, as shown in Figure 10a. The distance between the antisymmetric VIOs has no effect on the pressure drop between the mixer inlet and outlet, as shown in Figure 10b. This trend is similar to that observed in T-shaped micromixers with symmetric VIOs in the inlet channels.

**Figure 11:**
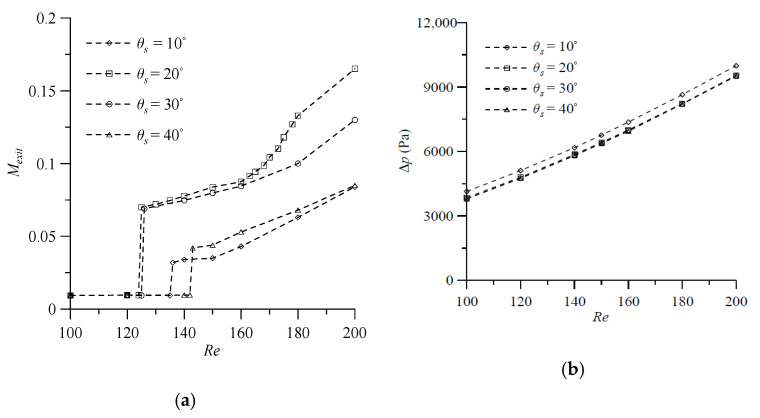
Effect of angle of attack $\theta_{s}$ on (**a**) the degree of mixing, and (**b**) the pressure drop in the mixers with *d* = 3*H*, *h* = 0.75*H*, *t* = 0.25*H* and $w_{d}=0.25H$ for various Reynolds numbers.

**Figure 12:**
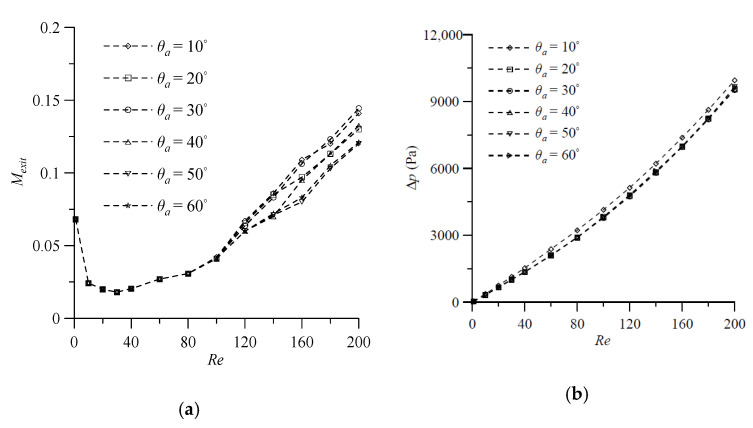
Effect of angle of attack $\theta_{a}$ on (**a**) the degree of mixing, and (**b**) the pressure drop in the mixers with *d* = 3*H*, *h* = 0.75*H*, *t* = 0.25*H* and $w_{d}=0.25H$ for various Reynolds numbers.

Next, efforts are made to investigate the effect of the angle of attack ($\theta_{s}$ or $\theta_{a}$) of the VIOs, which is illustrated in Figure 1c. The degrees of mixing at the outlet and the pressure drop between the mixer inlet and outlet for fluid flow in the T-shaped micromixers with symmetric VIOs at $\theta_{s}=$10°, 20°, 30° and 40° in the Reynolds number range from 100 to 200 are calculated, when the other geometrical parameters, d=3H, h=0.75H, t=0.25H and $w_{d}=0.25H$, are kept constant. The degrees of mixing of the cases with $\theta_{s}=$20° show the smallest value of *Re* corresponding to the transition from the vortex flow regime to the engulfment flow regime, as shown in Figure 11a. In the engulfment flow regime, the degree of mixing shows a maximum at $\theta_{s}=$20°, and then decreases with a further increase or decrease in the $\theta_{s}$ of the VIOs. When $W_{i}$, t and $w_{d}$ are fixed, the length of the VIOs decreases with the increase of $\theta_{s}$ [16]. Thus, the pressure drop decreases with the increase of $\theta_{s}$, as shown in Figure 11b. In addition, Figure 11b shows that the transition from the vortex flow regime and to the engulfment flow regime has no effect on the pressure drop. To investigate the influence of $\theta_{a}$, we consider a wider range of Reynolds number (1≤Re≤200) and the value of $\theta_{a}$ varies from 10° to 60°, with an interval of 10°. The simulation results for fluid mixing in T-shaped mixers with antisymmetric VIOs are examined for cases with the six values of $\theta_{a}$, d=3H, h=0.75H, t=0.25H and $w_{d}=0.25H$. Figure 12a shows that the degrees of mixing of the cases considered increase gradually as the *Re* increases beyond 30, and the effect of $\theta_{a}$ on fluid mixing becomes noticeable as the *Re* increases beyond 100. When *Re* > 100, one can see that the degree of mixing increases with the increase of $\theta_{a}$ for the low value of $\theta_{a}$, shows a maximum at $\theta_{a}=$ 30° and then decreases with further increase of $\theta_{a}$. The dependence of Δp on $\theta_{a}$ is similar to that of Δp on $\theta_{s}$, as shown in Figure 12b. Besides, the comparison of Figure 9b, Figure 10b, Figure 11b and Figure 12b reveals that the effect of $\theta_{s}$ or $\theta_{a}$ on Δp is greater than that of the distance between the antisymmetric VIOs on Δp.

**Figure 13:**
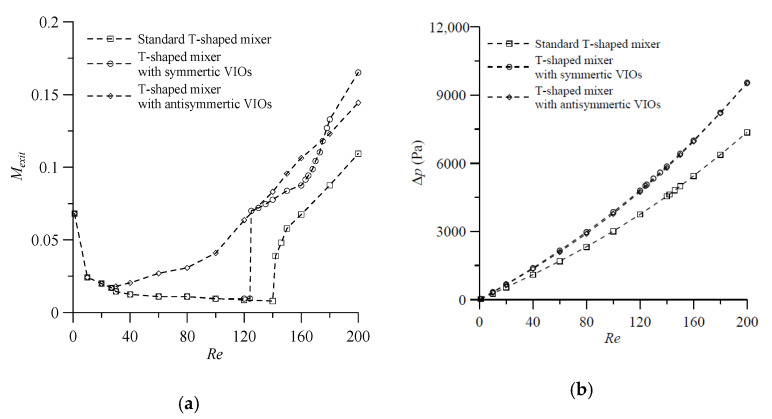
(**a**) Degree of mixing at the exit, and (**b**) pressure drop versus Reynolds number for the proposed mixers with VIOs and a standard planar T-shaped mixer.

As shown in the above study, the dimensions d=3H and $\theta_{s}=$20° for symmetric VIOs, and d=3H and $\theta_{a}=$ 30° for antisymmetric VIOs, are found to be optimum values for mixing performance. Further increase or decrease of these parameters negatively affected the degree of mixing. Figure 13a depicts the variation of the degrees of mixing of the proposed mixers predicted by using these parameter values and that of a standard planar T-shaped mixer over a wide range of *Re*. From Figure 13a, the following features may be noted. First, for the cases with low Reynolds numbers (Re<27), the effects of vortices are not strong enough to enhance mixing and the mixing relies mainly on diffusion in the three T-shaped mixers. For such cases, increasing the value of Re reduces the residence time and the mixing efficiency. Second, for the cases with Re≥27, the effects of the stretched contact surface between different fluids caused by antisymmetric VIOs gradually increases with the increase of *Re*, and so the enhancement of mixing by antisymmetric VIOs also gradually increases with the increase of *Re*. Meanwhile, fluid mixing in the other two T-shaped mixers still relies on diffusion and the mixing efficiency decreases with the increase of Re until, at the onset of the engulfment flow, the degree of mixing increases suddenly, which happens at Re≈125 in the T-shaped mixer with symmetric VIOs and at Re≈140 in the standard planar T-shaped mixer. Third, with the onset of the engulfment, the mixing efficiency in the T-shaped mixer with symmetric VIOs improved but is not better than that in the T-shaped mixer with antisymmetric VIOs for *Re* in the range 27–175. In addition, the increases of pressure drop caused by both types of VIOs mounted on the bottom of the inlet channels were almost equal and the effect increased with the increase of *Re*, as shown in Figure 13b.

## 4. Conclusions

A new approach for inlet flow modification by adding obstacles protruding into the flow at an angle of attack in the inlet channels of a T-shaped micromixer was proposed and investigated in this work. The dimensions d=3H and $\theta_{s}=$20° for symmetric VIOs, and d=3H and $\theta_{a}=$ 30° for antisymmetric VIOs, were found to be optimum values for mixing performance. Comparisons of the mixing performance of the proposed mixers with the selected values of geometric parameters of VIOs and that of a standard T-shaped mixer without VIOs showed that the antisymmetric VIOs in the inlet channels induced the stretched contact surface between different fluids. The enhancement of mixing by the VIOs gradually increased with the increase of *Re* for the cases with Re≥27, fluid mixing in the other two T-shaped mixers still relied on diffusion and the mixing efficiency decreased with the increase of Re until the onset of the engulfment flow, which happened at Re≈125 in the T-shaped mixer with symmetric VIOs and at Re≈140 in the standard planar T-shaped mixer. With the onset of the engulfment, the degree of mixing increased suddenly and so the mixing efficiency improved. For Re>175, the mixing efficiency of the T-shaped mixer with symmetric VIOs was even better than that in the T-shaped mixer with antisymmetric VIOs.

In summary, the early initiation of vortices in the inlet streams of a T-shaped micromixer by either symmetric or antisymmetric VIOs in the inlet channels can lead to improvements in mixing efficiency. The simple but effective geometrical modification can be combined with various geometric modifications of the mixing channel to enhance fluid mixing, and such hybrid micromixers will be considered in future work.

## References

[1] Nguyen N.T., Wu Z. (2005). Micromixers—A review. J. Micromech. Microeng..

[2] Lee C.-Y., Wang W.-T., Liu C.-C., Fu L.-M. (2016). Passive mixers in microfluidic systems: A review. Chem. Eng. J..

[3] Raza W., Hossain S., Kim K.Y. (2020). A review of passive micromixers with a comparative analysis. Micromachines.

[4] Gobby D., Angeli P., Gavriilidis A. (2001). Mixing characteristics of T-type microfluidic mixers. J. Micromech. Microeng..

[5] Engler M., Kockmann N., Kiefer T., Woias P. (2004). Numerical and experimental investigations on liquid mixing in static micromixers. Chem. Eng. J..

[6] Hoffmann M., Schlüter M., Räbiger N. (2006). Experimental investigation of liquid-liquid mixing in T-shaped micro-mixers using μ-LIF and μ-PIV. Chem. Eng. Sci..

[7] Hussong J., Lindken R., Pourquie M., Westerweel J., Ellero M., Hu X., Fröhlich J., Adams N. (2009). Numerical study on the flow physics of a T-shaped micro mixer. IUTAM Symposium on Advances in Micro- and Nanofluidics.

[8] Soleymani A., Yousefi H., Turunen I. (2008). Dimensionless number for identification of flow patterns inside a T-micromixer. Chem. Eng. Sci..

[9] Fani A., Camarri S., Salvetti M.V. (2013). Investigation of the steady engulfment regime in a three-dimensional T mixer. Phys. Fluids.

[10] Andreussi T., Galletti C., Mauri R., Camarri S., Salvetti M.V. (2015). Flow regimes in T-shaped micro-mixers. Comput. Chem. Eng..

[11] Galletti C., Roudgar M., Brunazzi E., Mauri R. (2012). Effect of inlet conditions on the engulfment pattern in a T-shaped micro-mixer. Chem. Eng. J..

[12] Schikarski T., Trzenschiok H., Peukert W., Avila M. (2019). Inflow boundary conditions determine T-mixer efficiency. React. Chem. Eng..

[13] Karvelas E., Liosis C., Benos L., Karakasidis T., Sarris I. (2019). Micromixing efficiency of particles in heavy metal removal processes under various inlet conditions. Water.

[14] Camarri S., Mariotti A., Galletti C., Brunazzi E., Mauri R., Salvetti M.V. (2020). An overview of flow features and mixing in micro t and arrow mixers. Ind. Eng. Chem. Res..

[15] Lin Y.C., Chung Y.C., Wu C.Y. (2007). Mixing enhancement of the passive microfluidic mixer with J-shaped baffles in the tee channel. Biomed. Microdevices.

[16] Hsiao K.-Y., Wu C.-Y., Huang Y.-T. (2014). Fluid mixing in a microchannel with longitudinal vortex generators. Chem. Eng. J..

[17] Vijayendran R.A., Motsegood K.M., Beebe D.J., Leckband D.E. (2003). Evaluation of a three-dimensional micromixer in a surface-based biosensor. Langmuir.

[18] Jiang F., Drese K.S., Hardt S., Küpper M., Schönfeld F. (2004). Helical flows and chaotic mixing in curved micro channels. AIChE J..

[19] Gigras A., Pushpavanam S. (2008). Early induction of secondary vortices for micromixing enhancement. Microfluid. Nanofluidics.

[20] Wu C.-Y., Tsai R.-T. (2013). Fluid mixing via multidirectional vortices in converging-diverging meandering microchannels with semi-elliptical side walls. Chem. Eng. J..

[21] Johnson T.J., Ross D., Locascio L.E. (2002). Rapid microfluidic mixing. Anal. Chem..

[22] Stroock A.D., Dertinger S.K.W., Ajdari A., Mezic I., Stone H.A., Whitesides G.M. (2002). Chaotic mixer for microchannels. Science.

[23] Branebjerg J., Gravesen P., Krog J.P., Nielsen C.R. Fast mixing by lamination. Proceedings of the Ninth International Workshop on Micro Electromechanical Systems.

[24] Schönfeld F., Hessel V., Hofmann C. (2004). An optimised split-and-recombine micro-mixer with uniform ‘chaotic’ mixing. Lab Chip.

[25] Hossain S., Kim K.Y. (2014). Mixing analysis of passive micromixer with unbalanced three-split rhombic sub-channels. Micromachines.

[26] Xia H.M., Wan S.Y.M., Shu C., Chew Y.T. (2005). Chaotic micromixers using two-layer crossing channels to exhibit fast mixing at low Reynolds numbers. Lab Chip.

[27] Lee J., Kwon S. (2009). Mixing efficiency of a multilamination micromixer with consecutive recirculation zones. Chem. Eng. Sci..

[28] Chen J.J., Lai Y.R., Tsai R.T., Lin J.D., Wu C.-Y. (2011). Crosswise ridge micromixers with split and recombination helical flows. Chem. Eng. Sci..

[29] Raza W., Kim K.Y. (2019). Asymmetrical split-and-recombine micromixer with baffles. Micromachines.

[30] Kockmann N., Engler M., Föll C., Woias P. Liquid mixing in static micro-mixers with various cross sections. Proceedings of the First International Conference on Microchannels and Minichannels.

[31] Clark J., Kaufman M., Fodor P.S. (2018). Mixing Enhancement in Serpentine Micromixers with a Non-Rectangular Cross-Section. Micromachines.

[32] Sultan M.A., Fonte C.P., Dias M.M., Lopes J.C.B., Santos R.J. (2012). Experimental study of flow regime and mixing in T-jets mixers. Chem. Eng. Sci..

[33] Sultan M.A., Pardilho S.L., Brito M.S.C.A., Fonte C.P., Dias M.M., Lopes J.C.B., Santos R.J. (2019). 3D mixing dynamics in t-jet mixers. Chem. Eng. Technol..

[34] Cortes-Quiroz C.A., Azarbadegan A., Zangeneh M. Characterization and optimization of a three dimensional T-type micromixer for convective mixing enhancement with reduced pressure loss. Proceedings of the ASME 2010 8th International Conference on Nanochannels, Microchannels and Minichannels.

[35] Ansari M.A., Kim K.-Y., Anwar K., Kim S.M. (2012). Vortex micro T-mixer with non-aligned inputs. Chem. Eng. J..

[36] Matsunaga T., Nishino K. (2014). Swirl-inducing inlet for passive micromixers. RSC Adv..

[37] Chen C., Zhao Y., Wang J., Zhu P., Tian Y., Xu M., Wang L., Huang X. (2018). Passive mixing inside microdroplets. Micromachines.

[38] Ou J., Moss G., Rothstein J. (2007). Enhanced mixing in laminar flows using ultrahydrophobic surfaces. Phys. Rev. E..

[39] Liosis C., Karvelas E.G., Karakasidis T., Sarris I.E. (2020). Numerical study of magnetic particles mixing in waste water under an external magnetic field. J. Water Supply Res. T..

[40] Shah R.K., London A.L. (1978). Laminar Flow Forced Convection in Ducts: A Source Book for Compact Heat Exchanger Analytical Data.

[41] Rani S.A., Pitts B., Steward P.S. (2005). Rapid diffusion of fluorescent tracers into staphylococcus epidermidis biofilms visualized by time lapse microscopy. Antimicrob. Agents Chemother..

[42] Hoffman J.D. (1993). Numerical Methods for Engineers and Scientists.

[43] Matsunaga T., Lee H.-J., Nishino K. (2013). An approach for accurate simulation of liquid mixing in a T-shaped micromixer. Lab Chip.

[44] Kuo M.-Y., Wu C.-Y., Hsu K.-C., Chang C.-Y., Jiang W. (2019). Numerical investigation of high-Peclet-number mixing in periodically-curved microchannel with strong curvature. Heat Transf. Eng..

[45] Szymczak P., Ladd A.J.C. (2003). Boundary conditions for stochastic solutions of the convection-diffusion equation. Phys. Rev. E.

[46] Bothe D., Bockhorn H., Mewes D., Peukert W., Warnecke H.J. (2010). Evaluating the quality of a mixture: Degree of homogeneity and scale of segregation. Micro and Macro Mixing: Analysis, Simulation and Numerical Calculation.

